# Translational selection in human: more pronounced in housekeeping genes

**DOI:** 10.1186/1745-6150-9-17

**Published:** 2014-07-10

**Authors:** Lina Ma, Peng Cui, Jiang Zhu, Zhihua Zhang, Zhang Zhang

**Affiliations:** 1 CAS Key Laboratory of Genome Sciences and Information, Beijing Institute of Genomics, Chinese Academy of Sciences, No.1 Beichen West Road, Chaoyang District, Beijing 100101, China; 2 Division of Chemical and Life Sciences & Engineering, King Abdullah University of Science and Technology, Jeddah, Thuwal 23955-6900, Kingdom of Saudi Arabia; 3 Department of Pathology, Massachusetts General Hospital and Harvard Medical School, Boston, Massachusetts 02114, USA

**Keywords:** Translational selection, Codon usage bias, Expression regulation, Housekeeping gene, Tissue-specific gene

## Abstract

**Background:**

Translational selection is a ubiquitous and significant mechanism to regulate protein expression in prokaryotes and unicellular eukaryotes. Recent evidence has shown that translational selection is weakly operative in highly expressed genes in human and other vertebrates. However, it remains unclear whether translational selection acts differentially on human genes depending on their expression patterns.

**Results:**

Here we report that human housekeeping (HK) genes that are strictly defined as genes that are expressed ubiquitously and consistently in most or all tissues, are under stronger translational selection.

**Conclusions:**

These observations clearly show that translational selection is also closely associated with expression pattern. Our results suggest that human HK genes are more efficiently and/or accurately translated into proteins, which will inevitably open up a new understanding of HK genes and the regulation of gene expression.

**Reviewers:**

This article was reviewed by Yuan Yuan, Baylor College of Medicine; Han Liang, University of Texas MD Anderson Cancer Center (nominated by Dr Laura Landweber) Eugene Koonin, NCBI, NLM, NIH, United States of America Sandor Pongor, International Centre for Genetic Engineering and biotechnology (ICGEB), Italy.

## Background

The unequal usage of synonymous codons, often termed as codon usage bias (CUB), is generally thought to be an intricate combined outcome of mutation pressure, natural selection, and genetic drift [1-5]. Although there are multiple different factors closely associated with CUB, including transcriptional selection [6,7], transcription-coupled mutation [8], biased gene conversion [8,9], exon splicing [10], mRNA structure and stability [11-13], gene function [14,15], biased codon usage is believed to stem from selection for translational efficiency and/or accuracy [16-18], implying that highly biased codon usage is correlated with high expression level. As the effectiveness of such selection on synonymous codon usage is also influenced by effective population size, translational selection is expected to be stronger in species with large populations.

Consistently, translational selection has been widely documented in prokaryotes and unicellular eukaryotes, as typified by *Escherichia coli*[19-22] and *Saccharomyces cerevisiae*[22-24] in which CUB correlates closely with gene expression level. In small populations, contrastingly, translational selection is not well explored; for example, it has been debated for time about whether translational selection does not exist or is too weak to be detectable in human [7,25-28], which is also complicated by heterogeneous composition isochores [29,30].

A sensitive CUB index is quite important in detecting translational selection, especially in species influenced by isochore effect. Unlike extant indexes, the CDC (Codon Deviation Coefficient) index effectively considers background nucleotide compositions and does not require any prior knowledge of reference gene sets [22]. As testified on empirical data, CUB values measured by CDC correlate positively with gene expression level, much better than *Nc*’, *Nc* (Effective Number of Codons), and CAI (Codon Adaptation Index) [22]. Recently, Doherty and McInerney utilized the CDC index, and demonstrated that translational selection frequently overcomes genetic drift to operate weakly in human and other vertebrates [31], suggesting that translational selection is a widespread mechanism, not confined to prokaryotes and unicellular eukaryotes.

To date, many studies have provided evidence for differential translational selection between highly and lowly expressed genes in human [27,31,32]. As different human genes undergo various selective pressures in association with their expression patterns [33], we wonder whether expression pattern, aside from expression level, plays a role in shaping heterogeneity of translational selection acting on human genes. The high-throughput sequencing technology enables generation of high-resolution transcriptome data, making it possible to systematically explore the difference of codon usage in human genes by considering both expression level and expression breadth. As it has been suspected that genes in multicellular organisms have too many expression constraints to achieve an analog codon choice pattern for the whole genome or for most of the expressed genes [17], we hypothesize that translational selection is more pronounced in human genes that are expressed ubiquitously and consistently in most or all tissues.

## Results and discussion

**Figure 1:**
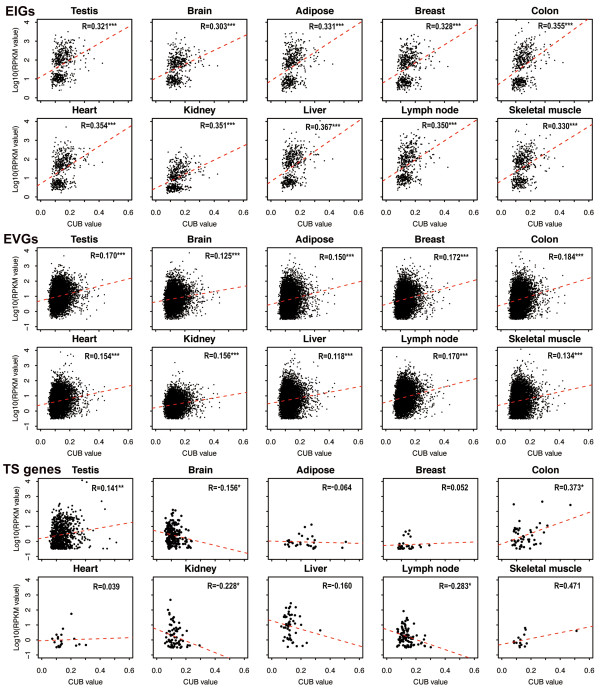
**Relationship between CUB and gene expression level.** Linear correlation (Pearson correlation) analyses between CUB and gene expression level (log 10 RPKM) were conducted in EIGs (expression-invariable genes)**,** EVGs (expression-variable genes), and TS (tissue-specific) genes across 10 human tissues. Linear correlation coefficient (R) is shown in each panel and *P*-value (F-test) is indicated by ‘*’ <0.05, ‘**’ <10^-3^, and ‘***’ <10^-10^. 505 EIGs, 7631 EVGs, 771 testis-specific genes, 192 brain-specific genes, 29 adipose-specific genes, 28 breast-specific genes, 47 colon-specific genes, 19 heart-specific genes, 77 kidney-specific genes, 51 liver-specific genes, 97 lymph node-specific genes, 15 skeletal muscle-specific genes, whose *P*-values for CUBs are less than 0.05, are used to draw the plots.

To address this hypothesis, we collected RNA-Seq data for 10 human tissues (see Methods) and defined housekeeping (HK) and tissue-specific (TS) genes (Additional file 1: Table S1) based on our previous studies [34,35]. Specifically, HK genes that are involved in the maintenance of basal cellular functions are expected to possess constant expression levels under a wide diversity of conditions. Accordingly, HK genes are defined strictly as expression-invariable genes (EIG; 613 genes) that are expressed in all the 10 tissues at relatively consistent expression levels, whereas expression-variable genes (EVG; 8,590 genes) are expressed in all 10 tissues at diverse expression levels. Similarly, a gene is defined strictly to be TS if it is expressed only in one tissue. As expected, EIGs, EVGs, and TS genes yield distinct correlation coefficients between CUB and gene expression level across all 10 tissues. Overall, the correlation is significantly positively stronger in EIGs than in EVGs or TS genes based on both Pearson (Figure 1) and Spearman correlation (Additional file 2: Table S2) analyses. We further classified genes into different groups according to their CUB values and demonstrated that the increase of gene expression level with CUB is more striking in EIGs by comparison to EVGs and TS genes (Additional file 3: Figure S1).

We also took account of variable expression breadths when investigating the correlation between CUB and gene expression level. Considering the 10 tissues examined here, we found that genes with narrow expression breadth (ranging from 1 to 8) do not tend to show significant positive correlation between CUB and expression level (Additional file 4: Table S3). By contrast, CUB does exhibit significant positive correlation with expression level when genes are expressed at broader breadths (that is, 9 and 10; Additional file 4: Table S3). Consistently, genes with expression breadth = 10, a mixture of EIGs and EVGs, present intermediate correlation coefficients, higher than EVGs and lower than EIGs (Figure 1 and Additional file 4: Table S3). Clearly, these results demonstrate that expression breadth is an important factor associated with translational selection, which is likely to operate more strongly on genes that are expressed ubiquitously in nearly all tissues.

**Figure 2:**
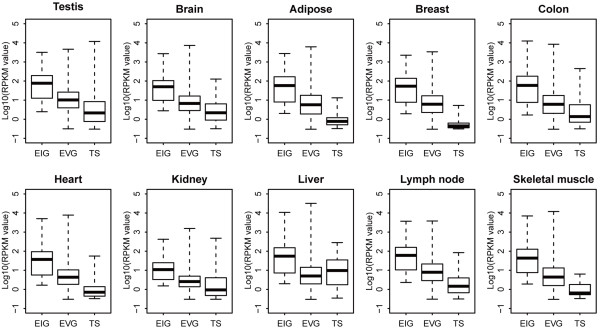
**Expression levels of EIGs (HK genes), EVGs, and TS genes in each tissue.** Box-plot is used to show the distribution of expression level (log 10 RPKM) based on RPKM values. The boxes depict data between the 25th and 75th percentiles with central horizontal lines representing the median values.

**Figure 3:**
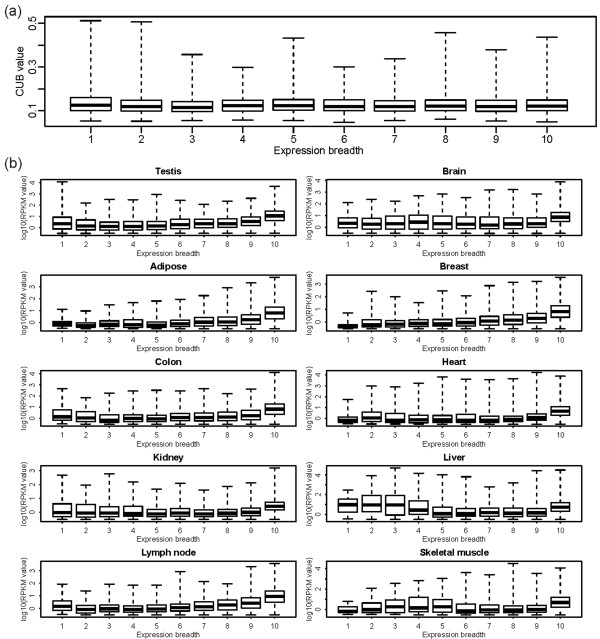
**CUB and expression level distributions considering different expression breadths. (a)** CUB distribution of genes that are expressed at different breadths. **(b)** Expression levels of genes that are expressed at different breadths in each tissue.

Evidence has accumulated that translational selection operates significantly in highly expressed genes in human [27,31,32]. As EIGs always exhibit higher expression levels than EVGs and TS genes in all examined tissues (Figure 2), we further investigated whether the stronger correlation between CUB and expression level in EIGs is caused by high expression only. We ranked EVGs according to expression level (that is, the highest RPKM among the 10 tissues for each gene) and performed the analysis based on the top 600, 1000, and 1300 highly expressed EVGs, as these three groups present significantly higher expression levels than EIGs (Wilcoxon test, *P* < 10^-10^), albeit the top 1300 EVGs have comparable median expression levels (RPKM = 148) with EIGs (RPKM = 145). Intriguingly, all these three groups exhibit weaker positive correlations (Pearson correlation) between CUB and gene expression level (R = 0.143, 0.137, and 0.147, respectively) than EIGs (R = 0.321). Therefore, high expression is not the only reason for stronger correlation between CUB and gene expression level in EIGs. When considering diverse expression breadths, for instance, in liver, genes with breadth 2 or 3 have comparable high expression levels with genes with breadth 10 (Wilcoxon test, *P* > 0.05) (Figure 3), but do not present significant positive correlation between CUB and gene expression level (Additional file 4: Table S3). Additionally, in adipose, genes with breadth 4, possessing significantly lower levels than those with breadth 9 (Wilcoxon test, *P* < 10^-10^) (Figure 3), exhibit stronger positive correlation between CUB and gene expression level (Additional file 4: Table S3). Taken together, these results indicate that high expression is not the only reason for stronger correlation between CUB and gene expression level in human.

Meanwhile, we observed that EIGs always exhibit higher expression levels than EVGs in all examined tissues (Figure 2), and consistently present larger CUBs than EVGs (Wilcoxon test, *P* < 10^-16^). When we used the highest RPKM among the 10 tissues to represent the expression level for each EIG or EVG, EIGs are still significantly higher in expression level than EVGs (Wilcoxon test, *P* < 10^-10^). Thus, we examined whether large CUBs in EIGs are attributable to high expression levels and smaller CUBs in EVGs are attributable to lower expression levels. We compared the top 400 highly expressed EIGs against the top 600 EVGs, as they have very similar median expression levels (median RPKM = 266 and 269, respectively). Yet, the top 400 EIGs present significantly lower expression level than the top 600 EVGs (Wilcoxon test, *P* < 0.05), and strikingly, have significantly larger CUB than the top 600 EVGs (Wilcoxon test, *P* < 10^-3^). This result indicates that EIGs exhibit higher expression than EVGs is not only because that EIGs present larger CUBs. Although gene expression level correlates closely with expression breadth, CUBs are comparable among different expression breadths (Figure 3). Therefore, high expression level does not necessarily correspond to large CUB in human, which suggests that CUBs in different expression patterns form under different mechanisms. In EIGs, CUBs are most likely to be closely associated with translational selction.

If translational selection is stronger in one gene, codon usage in this gene is more biased toward optimal codons (match the most abundant tRNAs or tRNAs that can be modified by ADAT (tRNA-dependent adenosine deaminases) [36]). We calculated frequencies of synonymous codons using the Relative Synonymous Codon Usage (RSCU) [37] to compare synonymous codon usage preferences for 17 amino acids (exclusive of three amino acids, viz., Met and Trp with each containing only one encoding codon, and Glu that corresponds to two tRNAs with equal count). We did find that in EIGs, high CUB genes are more biased in using optimal codons than low CUB genes (RSCU values of optimal codons are larger in high CUB genes than low CUB genes in most cases and high CUB genes preferentially use more optimal codons; Additional file 5: Table S4). In combination with the significantly positive correlation between CUB and gene expression level in EIGs (Figure 1), these results indicate that high CUB EIGs are under stronger translational selection than low CUB EIGs. To compare the strength of translational selection between EIGs, EVGs, and TS genes, we then examined the usage of optimal codons in the three groups.

**Figure 4:**
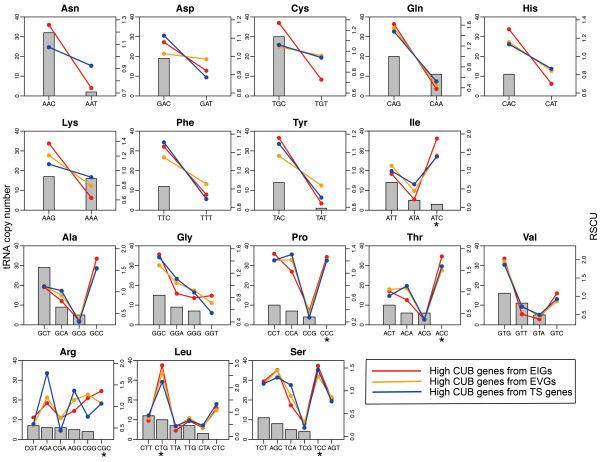
**Relative synonymous codon usage in high CUB genes.** tRNA copy number is shown on the left y-axis (grey bars) and RSCU (Relative Synonymous Codon Usage) is on the right y-axis (lines). Optimal codons (indicated by asterisk) correspond to the most abundant tRNAs or tRNAs that can be modified by ADAT (tRNA-dependent adenosine deaminases).

High CUB genes from these three groups show highly correlated patterns with codon usage, but EIGs are extremely biased in using optimal codons. First, in most cases, optimal codons’ RSCUs are larger in EIGs than in EVGs or TS genes. In other words, high CUB genes from EIGs tend to use optimal codons more frequently than those from EVGs or TS genes (Figure 4 and Additional file 5: Table S4). Specifically, high CUB genes from EIGs use optimal codons more frequently than those from EVGs in 16 amino acids (Asn, Asp, Cys, Gln, His, Lys, Phe, Tyr, Ile, Gly, Pro, Thr, Val, Arg, Leu, Ser) and than those from TS genes in 14 amino acids (Asn, Cys, Gln, His, Lys, Tyr, Ile, Gly, Pro, Thr, Val, Arg, Leu, Ser). Second, high CUB genes from EIGs preferentially utilize the optimal codons among all the degenerate codons in 15 amino acids (Asn, Asp, Cys, Gln, His, Lys, Phe, Tyr, Ile, Gly, Thr, Val, Arg, Leu, Ser), larger than those from EVGs (13 in count; Asn, Asp, Cys, Gln, His, Lys, Phe, Tyr, Ile, Gly, Thr, Val, Leu) or TS genes (14 in count; Asn, Asp, Cys, Gln, His, Lys, Phe, Tyr, Ile, Gly, Thr, Val, Leu, Ser) (Figure 4 and Additional file 5: Table S4). It should be noted, however, that high CUB genes from EIGs are not significantly larger in terms of CUB values than those from EVGs or TS genes (Wilcoxon test, *P* > 0.05). We also performed similar comparisons in each tissue and found that high CUB genes from EIGs use optimal codons more frequently and preferentially than high CUB TS genes in most tissues (Additional file 5: Table S4). Therefore, these results suggest that highly biased codon usage tends to correlate with stronger translational selection in EIGs, but may not function effectively to improve translational efficiency and/or accuracy in EVGs or TS genes.

As noticed, not all optimal codons are most highly used in human genes, which is probably because of weak correspondence of tRNA copy number with tRNA abundance in human [7], or presumably due to complex balance of codon usage between optimal codons and non-optimal codons, for example, to reach high translational efficiency [38], affect protein expression, structure and function [39], or achieve circadian clock conditionality [15]. Interestingly, a total of 15 optimal codons (AAC, GAC, TGC, CAG, CAC, AAG, TTC, TAC, GGC, GTG, *ATC*, *ACC*, *CGC*, *CTG*, and *TCC*; codons in italic are optimal codons that are associated with ADAT modification [36]) that are preferentially used by high CUB genes in EIGs, are all GC-ending (Figure 4). We did find that EIGs exhibit significant linear positive correlation between GC3 and CUB (Additional file 6: Figure S2a), high CUB genes are significantly higher in GC3 than low CUB genes (Additional file 6: Figure S2b), and GC3 shows evident and significant linear correlation with gene expression level (R = 0.513, *P* < 10^-10^). It is indicated that, in addition to translational selection, other factors that correlate with GC3, e.g. transcriptional selection [40,41], mRNA stability [11,12], biased gene conversion [8,9], may also have combined with translational selection to contribute to the positive correlation between CUB and gene expression level. As translational regulation rather than transcriptional regulation or mRNA stability is more pronounced in influencing protein level in mammals [42], future investigations might as well involve protein expression data to verify such strong translational selection in human HK genes and take account of translation initiation [13] and elongation as well as codon order [43].

## Conclusions

To sum up, these results are, to our knowledge, the first to indicate that in human HK genes that are expressed ubiquitously and consistently in most or all tissues are still under significant translational selection, more pronounced than EVGs and TS genes. Although there are multiple intricate mechanisms complicating the regulation of human gene expression in contrast to prokaryotes and unicellular eukaryotes, translational selection is likely a fundamental mechanism to be universally operative in a diversity of species, acting more significantly remarkable in human HK genes. Given that HK genes are more ancient than TS genes [44], such consistency between the strength of translational selection and the evolutionary age of genes that is related to gene function and evolutionary constraint suggests that selection strength on synonymous codon usage might provide a proxy for estimate of gene age [45]. Most importantly, such stronger translational selection in HK genes indicates that when human genes are transcribed at similar high levels, HK genes are likely to be more efficiently and/or accurately translated into proteins, which will inevitably open up a new understanding of HK genes and the regulation of gene expression.

## Methods

We collected RNA-Seq data from 10 human tissues (testis, brain, adipose, colon, heart, breast, kidney, liver, lymph node, and skeletal muscle) [46], mapped them onto the human genome sequence, and calculated RPKM values using the methods previously described [34,35]. We retrieved gene sequences of *Homo sapiens* (human; hg19) from UCSC and adopted our newly-developed measure—Codon Deviation Coefficient (CDC) [22], to estimate CUBs for all examined genes. CUBs with *P*-value less than 0.05 were used. Genes were classified into five groups according to their CUB values: low (<0.10), medium-low (0.10-0.13), medium (0.13-0.16), medium-high (0.16-0.19), and high (>0.19). The tRNA copy numbers for human were extracted from the genomic tRNA database (hg19) (GtRNAdb; http://gtrnadb.ucsc.edu/Hsapi19/Hsapi19-summary-codon.html) [47].

## Reviewer’s comments on the original manuscript

### Reviewer 1

Yuan Yuan, Baylor College of Medicine

Han Liang, University of Texas MD Anderson Cancer Center (nominated by Dr Laura Landweber)

Quality of written English:Acceptable

In this study, Ma et al. examined the translational selection in human by comparing the codon usage bias (CUB) across housekeeping genes (HK), expression-variable genes (EVG) and tissue-specific genes (TS). Overall, it is an interesting question. However, to make a solid conclusion that “translational selection is closely associated with expression pattern”, some improvements should be made, including providing more details about experimental design and results, and justification of cutoffs.

Major comments:

1. Correct definition of different gene categories (HK, EVG, TS) lays the basis for this study. However, not enough detail was provided for how these categories were determined. For example, “relatively consistent expression levels” is a very vague description for defining HK genes. Could the authors present the criterion in a more specific way? How many TS genes were identified? Do these numbers/genes match with previously reported? These details should be provided.

*Response: Thanks for the comments and sorry for the unclear description. The definition of gene categories (EIG, EVG, TS) have been introduced in our previous papers (Cui et al. The transcript-centric mutations in human genomes. 2012; Cui et al. Distinct contributions of replication and transcription to mutation rate variation of human genomes, 2012). A gene is defined strictly to be TS if it is expressed only in one tissue. In order to identify EIGs and EVGs, we first isolated ubiquitously expressed genes (expressed in all the 10 human tissues) and divided them into 1,001 groups according to their expression levels to estimate the relative expression levels. We subsequently performed a hierarchical clustering analysis based on the relative expression levels. We then chose genes that are expressed in all the 10 tissues at relatively consistent expression levels and defined them as EIGs (Figure *1*B in Cui et al. The transcript-centric mutations in human genomes). The remaining ubiquitously expressed genes are defined as EVGs. In this manuscript, HK genes are defined strictly as EIGs.*

*We collected RNA-Seq transcriptome data to identify TS genes. Albeit strictly defined, 1,543 TS genes are obtained (920 in testis, 215 in brain, 31 in adipose, 32 in breast, 54 in colon, 21 in heart, 82 in kidney, 56 in liver, 117 in lymph node, and 15 in skeletal muscle;Additional file *1: *Table S1). Previous studies identified 198 TS genes from six tissues based on microarray data (Plotkin et al. Tissue-specific codon usage and the expression of human genes) and 2,126 TS genes from 18 tissues based on EST data (Semon et al. No evidence for tissue-specific adaptation of synonymous codon usage in humans). Therefore, considering the average count of TS genes in one tissue, our dataset of TS genes identified based on RNA-Seq is relatively more comprehensive. KEGG annotations indicate that functions of our TS genes are highly correlated with tissues’ functions (data not shown).*2. Lack of metrics/numerical/statistic summary for the statements claimed. “Overall, the correlation is significantly positively stronger in EIGs than in EVGs or TS genes (Figure 1).”: please provide the statistical tests (statistics, p-values) for “significantly positive stronger”. Please provide the gene number count (N = ?) for each case in Figure 1. In many cases for TS genes, the data seem too sparse to achieve a significant statistical difference. In this sense, the comparison between HK and TS genes may be not fair. Please provide new analysis accounting for size differences between different gene sets to justify the validity of current result.

Response: Thanks for pointing this out. P-value (F-test) is indicated by ‘*’ <0.05, ‘**’ <10^*-3*^, and ‘***’ <10^*-10*^*in Figure *1*(please refer to the legend of Figure *1*). Detailed information of TS genes is listed inAdditional file *1: *Table S1. Following your suggestions, the gene count for each case is added in the legend of Figure *1*. When examining the correlation between gene expression and CUB, we estimated correlation coefficients in HK and TS genes, respectively. We noticed the difference of sample sizes between HK and TS genes when comparing correlation coefficients between such two gene sets. In order to account for possible bias resulting from different sample sizes, what we often do is to randomly sample the same number from HK and TS genes and calculate the correlation coefficient based on this sampled set. This process should be repeated many times (e.g., 1000, for removing stochastic bias) and accordingly, a distribution of correlation coefficient can be obtained. To our experience, however, the more times we repeat, the closer the median of the distribution approaches the original one (data not shown). That is to say, our conclusion remains valid when comparing the correlation coefficients that are derived from two datasets with different sample size. In addition to linear correlation (Pearson correlation) analyses, we also performed Spearman correlation analyses to investigate the correlation relationship between CUB and gene expression level, which are summarized in* Additional file 2: *Table S2, and found that the correlation is indeed significantly positively stronger in EIGs than in EVGs or TS genes. We further performed RSCU comparisons and validated that translational selection is stronger in EIGs (Figure*4*,Additional file *5: *Table S4).*3. Many of the genes in Figure 1 had very low expression (RPKM <1). Since there are no replicates of samples or measurements in this study, the expression value for lowly expressed genes may be highly unreliable, and this concern should be considered.

Response: Yes, it is true that some genes, especially in TS genes, are lowly expressed. Our study is a completely bioinformatic work and all data used here were extracted from published papers in which replicates of samples or measurements have been already considered.

4. The authors claimed that “the increase of gene expression level with CUB is more striking in EIGs by comparison to EVGs and TS genes (Additional file 3: Figure S1)”. Although the median expression of EIG grouping by CUB show an increasing pattern visually, is this increase statistically significant (e.g., when comparing adjacent groups)? What is the number of genes in each group?

Response: We performed Wilcoxon tests between adjacent groups in EIGs. The P-values are, L vs. M-L, P = 0.0002; M-L vs. M, P = 0.15; M vs. M-H, P = 0.0006; M-H vs. H, P = 0.15. According to the P-values, only the comparisons between rank L and M-L, and between rank M and M-H are statistically significant (P < 0.05). Gene counts from rank Low to High are 3426, 4608, 3109, 1422, 1040 in all expressed genes, 59, 148, 134, 82, 82 in EIGs, 1932, 2665, 1749, 763, 522 in EVGs, and 171, 214, 164, 98, 124 in testis-specific genes.

5. Please provide the group sizes for Additional file 2: Table S2. Again, the size effect should be considered when comparing the group of genes with different expression breadth.

Response: Following your suggestion, the gene count for each group is added in the table. In addition to breadth 1, there are at least 30 genes in each group and most of the groups (90 %) have more than 100 genes. As responded above, the size effect is not an issue here.

6. Association does not imply causality. Even “EIGs always exhibit higher expression levels than EVGs and TS genes in all examined tissues”, high expression may not be the cause for the observation that “the stronger correlation between CUB and expression level in EIGs”. Therefore, “the only cause” is not suitable, and this limitation should be discussed.

Response: It is believed that highly expressed genes tend to use optimal codons more frequently to achieve high translation efficiency and accuracy, and thus exhibit highly biased codon usage. The higher the expression level, the larger the codon usage bias value is. Therefore, high expression level may cause a correlation between CUB and expression level.

7. It is not clear how the ranked EVGs were analyzed. Since the rank was based on the highest RPKM among the 10 tissues for each gene, was the analysis conducted in a tissue-specific way or pooled together? The choice of top 600, 1000 and 1300 highly expressed EVGs is not justified. Please provide more details on the analysis and the choice of the cutoffs.

Response: This analysis was conducted by pooling together all the expression values. As there are 613 EIGs, we chose similar number, 1.5 times, and 2 times the number of EIGs from EVGs.

Minor comments:

1. To make the manuscript more readable for a broader audience, please provide the full names of the abbreviations upon the first usage in the text, such as Nc, CAI, and GC3.

Response: Thanks for the suggestion. We have revised accordingly.

2. “HK” and “EIG” were used interchangeably in the text for housekeeping genes. Is it really necessary to use these two terms for the same context?

*Response: Most studies define HK genes as those that are ubiquitously expressed in most or all tissues and neglect the heterogeneity of expression level. According to our analysis, EIGs are most likely to be more efficiently and/or accurately translated into proteins. We use HK genes at the beginning and the end of our manuscript to emphasis that it is of basic biological significance to strictly define HK genes as EIGs.*3. Please describe how the RPKM value was normalized in Figure 2b.

*Response: We normalized RPKM values by considering that all the tissues’ total RPKM values are the same, e.g. equal to the total RPKM value of testis. Considering that this normalization makes little sense to the comparison within tissues, we removed Figure*2*b in the revised manuscript.*4. Please avoid using “always” when in fact there is some exception (e.g., Figure 4).

Response: Thanks for the suggestion. We have revised accordingly.

### Reviewer 2

Eugene Koonin, NCBI, NLM, NIH, United States of America

Report form:This paper addresses the long-standing problem of translational selection in mammalian genes and claims that translational selection in house-keeping genes is stronger that it is in tissue-specific genes. The problem certainly is of interest. However, I find the observation itself unsurprising and the analysis simplistic and limited in scope. To begin with, the plots in Figure 1 that compare the correlations between CUB and expression level tissue by tissue for different groups of genes do not look convincing at all. The authors show higher correlation coefficients for house-keeping compared to tissue-specific genes. However, all correlations are not particularly high, and there is no attempt to investigate how they are affected by outliers; it would have been more appropriate to use Spearman rather Pearson correlation. These comparisons are at least internally consistent. Things get more difficult when it comes to disentangling the dependences of the CUB on expression breadth and expression level. This would have been interesting, in principle, but I am not even sure what is the conclusion at the end. The article reports several comparisons, all quite convoluted. Moreover, the top plot in Figure 3 shows no dependence between expression breadth and CUB. So does such a dependence exist or not? I would further add that the authors limit all their analysis to humans whereas all the relevant data are available for mouse as well. A comparative analysis would have been much more convincing.

In general, I find the possibility that house-keeping genes in mammals are subject to stronger translational selection than tissue-specific genes to be quite believable and not even surprising. The magnitude of the difference and the comparative analysis of the effects of expression level and expression breadth would have been of interest but the paper as it stands makes a very weak attempt to address these issues.

Quality of written English: Needs some language corrections before being published

*Response: Following your comments, we additionally performed Spearman correlation analysis to investigate the correlation relationship between CUB and gene expression level (see details inAdditional file **2*: *Table S2), and found that the correlation is indeed significantly positively stronger in EIGs than in EVGs or TS genes.*

*According to Figure *3*, CUB does not change gradually along with expression breadth and CUB does not even tend to be different between different expression breadths. However, when genes are expressed at broader breadths (Additional file *4: *Table S3), CUB exhibits significant positive correlation with expression level. Therefore, although expression breadth is not correlated with CUB, it is an important factor associated with translational selection, which is likely to operate more strongly on genes that are expressed ubiquitously in nearly all tissues.*

*It is believed that high expression level may cause stronger correlation between gene expression level and CUB, and EIGs do exhibit higher expression level than EVGs and TS genes. The comparisons in Paragraph 3 in section “Results and discussion” show that in human, high expression is not the only reason for stronger correlation between CUB and gene expression level. Comparisons in Paragraph 4 in section “Results and discussion” indicate that CUBs in different expression patterns are formed under different mechanisms. Indeed, the high CUB genes in EIGs tend to use optimal codons more frequently (Paragraph 5 and 6 in section “Results and discussion”), leading to stronger correlation between CUB and gene expression level. In summary, the stronger correlation between CUB and gene expression level in EIGs is attributable to the combined effects of high expression level and the ubiquitous and consistent expression pattern.*

As mentioned in Background, it has been debated for time about whether translational selection does not exist or is too weak to be detectable in human. In this paper, we mainly investigated translational selection in human and elucidated that HK genes are most likely to be more efficiently and/or accurately translated into proteins. We sincerely appreciate you for these valuable comments and would like to investigate the universal of this finding in other species, such as mouse.

### Reviewer 3

Sandor Pongor, International Centre for Genetic Engineering and biotechnology (ICGEB), Italy

Report form:

Codon preferences are generally believed to emerge from a balance between mutational biases and natural selection via translational optimization. Fast-growing microbes like *Escherichia coli* or *Saccharomyces cerevisiae* have characteristic codon preferences that reflect the composition of their respective genomic tRNA pool. It is thought that optimal codons may help to achieve faster translation rates and high accuracy, and accordingly, translational selection is expected to be stronger in highly expressed genes. Machine learning methods detecting the effects of translational selection on the one hand, while controlling for local variation in nucleotide substitution patterns were used in the past to show evidence that translational selection in prokaryotes is practically universal and the characteristic differences in codon usage could be used for designing artificial genes of high expression. In the present paper, Ma and associates confirm that translational selection is closely associated with expression pattern of human genes. The results suggest that human housekeeping genes are better translated into proteins. The findings are clearly described.

Quality of written English:Acceptable

## Reviewers’ comments on the revised manuscript

### Reviewer 1

Yuan Yuan, Baylor College of Medicine

Han Liang, University of Texas MD Anderson Cancer Center (nominated by Dr Laura Landweber)

Report form:

The reviewer appreciates the authors’ efforts in addressing the original comments and concerns. The manuscript is now suitable for publication in Biology Direct.

Quality of written English:Acceptable

### Reviewer 2

Eugene Koonin, NCBI, NLM, NIH, United States of America

Report form:

In the revision, the authors provide some additional analysis and explanations. Unfortunately, this does not change things qualitatively. I still maintain that this work is, at best, a minor contribution to the existing understanding of translational selection.

Quality of written English:Needs some language corrections before being Published

Response: Thanks for the comments. Translational selection is more complex in human than in single-cellular organisms. We agree that evidences from large-scale data analysis and experimental verification have to be done to fully elucidate the regulation mechanisms of translational selection. We believe that our present work can provide hints for other related studies. As requested, we have proofed the manuscript accordingly.

### Reviewer 3

Sandor Pongor, International Centre for Genetic Engineering and biotechnology (ICGEB), Italy

Report form:

Modifications accepted

Quality of written English:Acceptable

## Abbreviations

HK gene: Housekeeping gene; TS gene: Tissue-specific gene; CUB: Codon usage bias; CDC: Codon deviation coefficient; Nc: Effective number of codons; CAI: Codon adaptation index; EIG: Expression-invariable gene; EVG: Expression-variable genes; GC3: GC content at the third codon position.

## Competing interests

The authors declare that they have no competing interests.

## Authors’ contributions

ZhaZ conceived of the study, and participated in its design and coordination and drafted the manuscript. LM participated in its design, and analyzed the data and drafted the manuscript. PC, JZ, ZhiZ participated in discussion and revised the manuscript. All authors read and approved the final manuscript.

## Authors’ information

LM: Ph.D., assistant research fellow at Beijing Institute of Genomics.

PC: Ph.D., research scientist at King Abdullah University of Science and Technology.

JZ: Ph.D., postdoctoral associate at Massachusetts General Hospital and Harvard Medical School.

ZhiZ: Ph.D., principal investigator at Beijing Institute of Genomics.

ZhaZ: Ph.D., principal investigator at Beijing Institute of Genomics.

## Supplementary Material

Additional file 1: Table S1 Gene list of EIGs, EVGs and TS genes. Click here for file

Additional file 2: Table S2 Spearman correlation analysis results between CUB and gene expression level. Click here for file

Additional file 3: Figure S1 Expression levels of human genes with different CUB ranks. Human genes are grouped into five ranks in terms of their CUB values: L: low (<0.10), M-L: medium-low (0.10-0.13), M: medium (0.13-0.16), M-H: medium-high (0.16-0.19), and H: high (>0.19). Distribution of gene expression level (log 10 RPKM) is shown as a box-plot in each gene group. Expression level of all expressed genes, EIGs, EVGs, and TS genes are all based on data of testis. The boxes depict data between the 25th and 75th percentiles with central horizontal lines representing the median values. Click here for file

Additional file 4: Table S3 Correlation between CUB and gene expression level considering different expression breadths. Click here for file

Additional file 5: Table S4 Relative synonymous codon usage values of different genes. Click here for file

Additional file 6: Figure S2 Relationship between CUB and GC compositions in EIGs. (a) Linear correlation (Pearson correlation) analyses between CUB and GC compositions. Correlation coefficient (R) is shown in each panel and *P*-value (F-test) is indicated by ‘*’ <0.05, ‘**’ <10^-3^, and ‘***’ <10^-10^. (b) Comparison of GC content between high CUB and low CUB groups. Wilcoxon tests were performed between high CUB and low CUB groups in EIGs. GC contents at three different codon positions are denoted as GC1, GC2, and GC3, respectively. *P* values: ‘*’ <0.05, ‘**’ <10^-3^, ‘***’ <10^-5^. Click here for file
