# Supplementary figures and images for: Translational selection in human: more pronounced in housekeeping genes
Source: Biol Direct. 2014 Jul 10;9:17. doi: 10.1186/1745-6150-9-17 (PMC4100034; doi:10.1186/1745-6150-9-17)

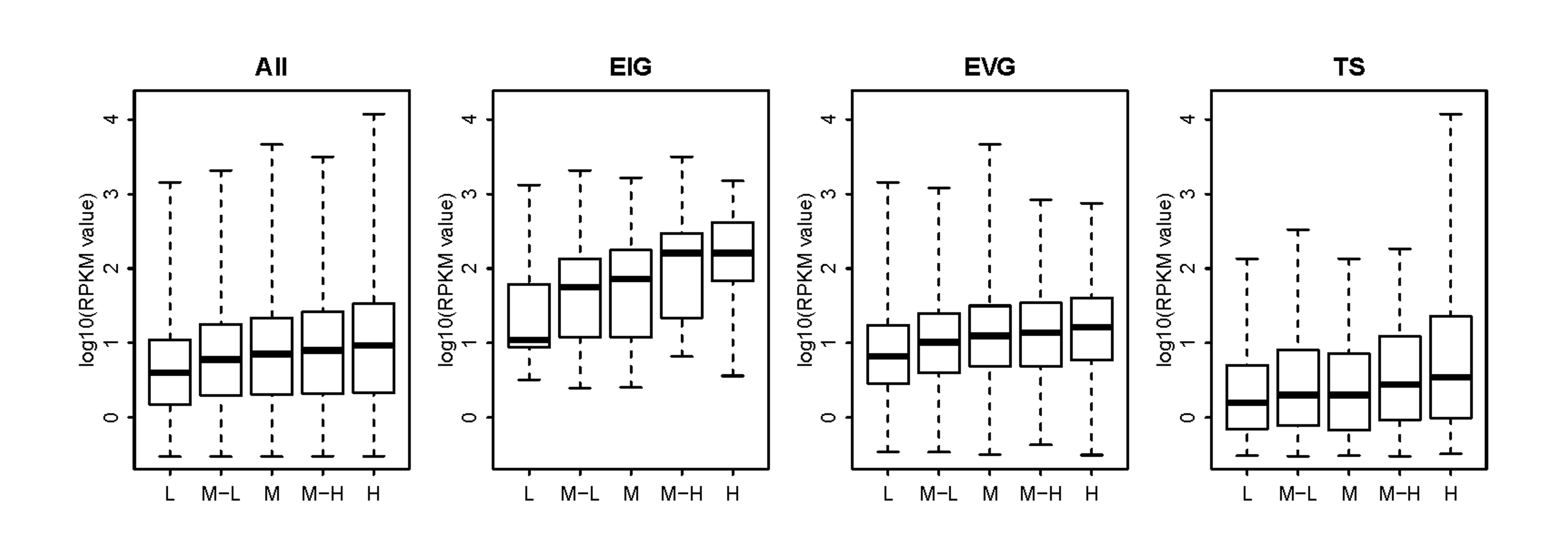

Supplement: Additional file 3: Figure S1 — Expression levels of human genes with different CUB ranks. Human genes are grouped into five ranks in terms of their CUB values: L: low (<0.10), M-L: medium-low (0.10-0.13), M: medium (0.13-0.16), M-H: medium-high (0.16-0.19), and H: high (>0.19). Distribution of gene expression level (log 10 RPKM) is shown as a box-plot in each gene group. Expression level of all expressed genes, EIGs, EVGs, and TS genes are all based on data of testis. The boxes depict data between the 25th and 75th percentiles with central horizontal lines representing the median values. [file 1745-6150-9-17-S3.tiff]

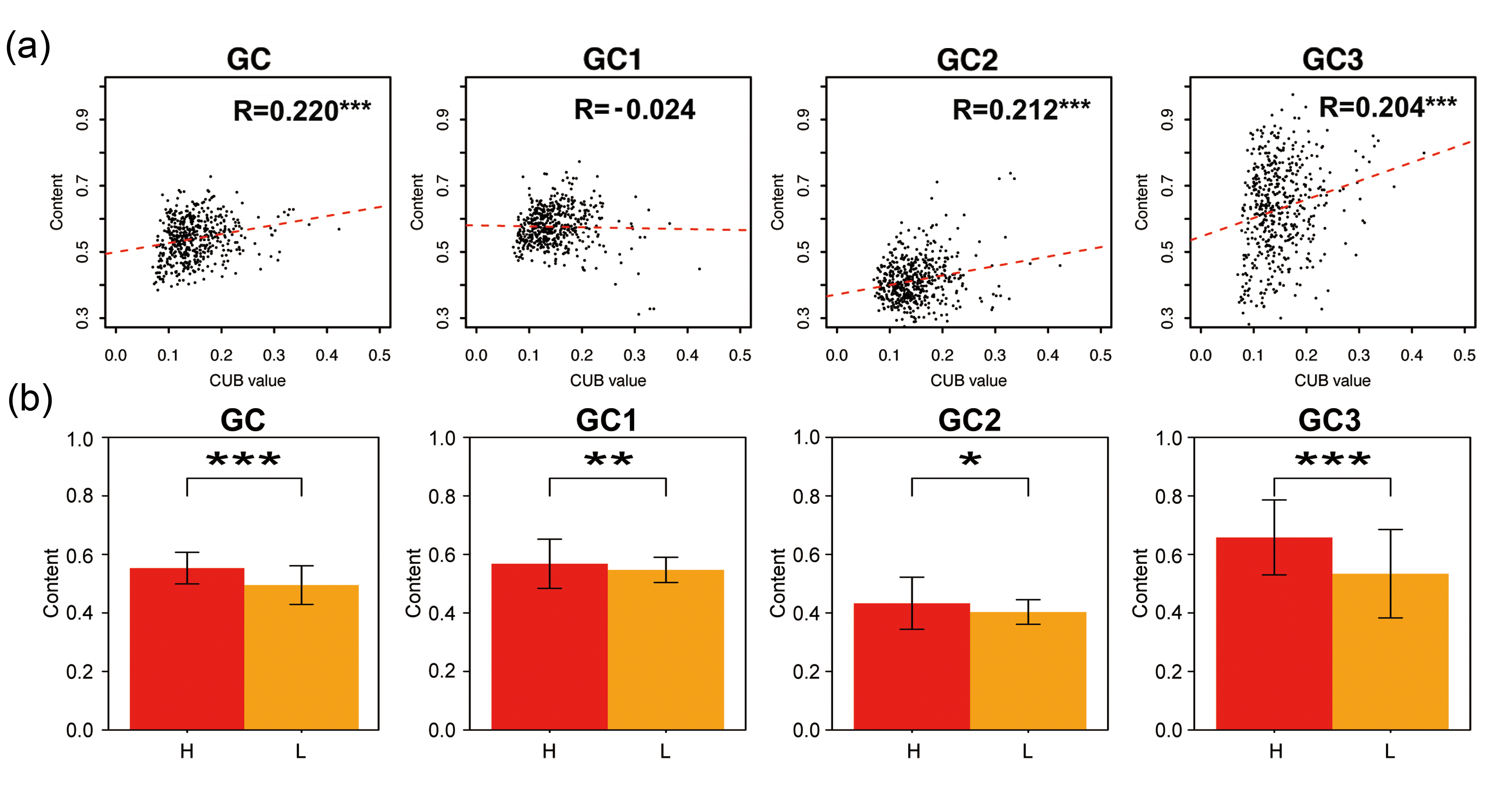

Supplement: Additional file 6: Figure S2 — Relationship between CUB and GC compositions in EIGs. (a) Linear correlation (Pearson correlation) analyses between CUB and GC compositions. Correlation coefficient (R) is shown in each panel and P-value (F-test) is indicated by ‘*’ <0.05, ‘**’ <10-3, and ‘***’ <10-10. (b) Comparison of GC content between high CUB and low CUB groups. Wilcoxon tests were performed between high CUB and low CUB groups in EIGs. GC contents at three different codon positions are denoted as GC1, GC2, and GC3, respectively. P values: ‘*’ <0.05, ‘**’ <10-3, ‘***’ <10-5. [file 1745-6150-9-17-S6.tiff]
